# Phylosymbiosis, diet and gut microbiome-associated metabolic disease

**DOI:** 10.1093/emph/eoaa019

**Published:** 2020-06-16

**Authors:** Elizabeth K Mallott, Katherine R Amato

**Affiliations:** e1 Department of Anthropology, Northwestern University, Evanston, IL, USA; e2 Department of Anthropology, Dartmouth College, Hanover, NH, USA

## DEFINITION AND BACKGROUND

**Figure 1.**
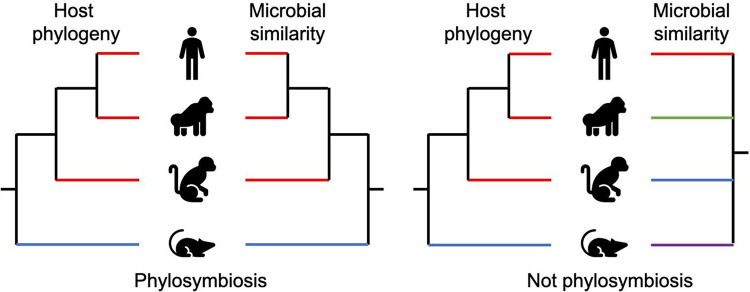
Phylosymbiosis versus stochastic assemblage

Microbiomes are influenced both by host evolutionary history and ecological context. Phylosymbiosis has been proposed as a framework to understand how evolutionary relationships among hosts predict the structure of the gut microbiome [1]. In this scenario, a hierarchical tree of similarities among host-associated microbiomes would mirror a phylogenetic tree of evolutionary relationships among hosts (Fig. 1). Phylosymbiosis can arise through coevolution and also through environmental, dietary or physiological similarities that correspond with shared evolutionary history.

Patterns of phylosymbiosis in the gut microbiome have been observed in multiple groups of animals [2]. However, this is but one eco-evolutionary process that shapes the composition of host-associated microbiomes. At times, host ecology plays a larger role in structuring microbial communities [3, 4]. There is mounting evidence that human-associated gut microbiomes do not necessarily follow a pattern of phylosymbiosis [4, 5]. Within the human microbiome, there are bacteria that have coevolved with their host, and others that have not [5].

## EXAMPLES IN CLINICAL MEDICINE AND PUBLIC HEALTH

Changes in the gut microbiome have been associated with metabolic diseases in humans, sparking interest in microbially based health interventions. While fecal microbial transplantation has worked in some specific situations (*Clostridium difficile* infection), results are less consistent in others, including for metabolic diseases [6]. The results of prebiotic and probiotic treatments have been similarly mixed. Because some microbes in our gut follow a pattern of phylosymbiosis, mirroring our evolutionary history, but others are environmentally acquired during an individual’s lifetime and do not follow a pattern of phylosymbiosis [4, 5], the success of microbially based health interventions likely depends on the specific eco-evolutionary processes influencing the microbial taxa involved.

Developing effective, targeted interventions requires understanding which bacterial taxa are associated with a metabolic disease and the evolutionary and ecological dynamics that determine the pathways by which those bacteria are acquired. Bacteria that are recently environmentally acquired may be good candidates for prebiotic or dietary interventions, while fecal microbial transplants may allow for reestablishment of taxa that are more closely linked to our evolutionary history but missing in an individual’s gut microbiome.

For example, short-chain fatty acids (SCFAs) are produced by the gut microbiome and used by the host for energy. One SCFA, butyrate, shows important associations with host metabolic health. In humans, decreased microbial butyrate production increases inflammation and risk of metabolic disease. Since many metabolic functions of the gut microbiome, such as SCFA production, are more strongly influenced by current human ecology than phylogenetic history [4], bacteria involved in butyrate fermentation are potentially acquired through interactions with our environment, not phylosymbiosis. Therefore, prebiotic or dietary interventions that target butyrate production may be more successful than fecal transplants.
